# Multidimensional Landscape of SA-AKI Revealed by Integrated Proteomics and Metabolomics Analysis

**DOI:** 10.3390/biom13091329

**Published:** 2023-08-30

**Authors:** Jiatong Xu, Jiaying Li, Yan Li, Xiaoxiao Shi, Huadong Zhu, Limeng Chen

**Affiliations:** 1 Emergency Department, State Key Laboratory of Complex Severe and Rare Diseases, Peking Union Medical College Hospital, Chinese Academy of Medical Science and Peking Union Medical College, Beijing 100730, China; xujiatong@pumch.cn (J.X.); liyan06@pumch.cn (Y.L.); 2 Medical Research Center, Peking Union Medical College Hospital, Chinese Academy of Medical Science and Peking Union Medical College, Beijing 100730, China; 3 Department of Nephrology, State Key Laboratory of Complex Severe and Rare Diseases, Peking Union Medical College Hospital, Chinese Academy of Medical Science and Peking Union Medical College, Beijing 100730, China; pumc_yingying@student.pumc.edu.cn (J.L.); shixiaoxiao@pumch.cn (X.S.)

**Keywords:** SA-AKI, proteomics, metabolomics, biomarker, mitochondrial dysfunction

## Abstract

Sepsis-associated acute kidney injury (SA-AKI) is a severe and life-threatening condition with high morbidity and mortality among emergency patients, and it poses a significant risk of chronic renal failure. Clinical treatments for SA-AKI remain reactive and non-specific, lacking effective diagnostic biomarkers or treatment targets. In this study, we established an SA-AKI mouse model using lipopolysaccharide (LPS) and performed proteomics and metabolomics analyses. A variety of bioinformatic analyses, including gene set enrichment analysis (GSEA), weighted gene co-expression network analysis (WGCNA), protein and protein interactions (PPI), and MetaboAnalyst analysis, were conducted to investigate the key molecules of SA-AKI. Integrated proteomics and metabolomics analysis revealed that sepsis led to impaired renal mitochondrial function and metabolic disorders. Immune-related pathways were found to be activated in kidneys upon septic infection. The catabolic products of polyamines accumulated in septic kidneys. Overall, our integrated analysis provides a multidimensional understanding of SA-AKI and identifies potential pathways for this condition.

## 1. Introduction

Sepsis is characterized as organ malfunction derived from the harmful reaction of a host to an infection. Sepsis-associated acute kidney injury (SA-AKI) is a severe and common complication of sepsis defined as an acute kidney injury (AKI) occurring in the presence of sepsis without any other significant contributing factors or characterized by the simultaneous presence of both Sepsis-3 and Kidney Disease Improving Global Outcomes (KDIGO) criteria [1,2]. The incidence of AKI is approximately 40–50% among septic patients [3]. Individuals suffering from AKI have an increased likelihood of progressing to chronic kidney disease and end-stage renal disease along with facing a higher long-term mortality rate following sepsis, making an early assessment of septic AKI probability and the accurate prediction of clinical outcomes crucial [4,5]. However, the pathogenesis of SA-AKI is complex, involving various factors such as inflammation, microvascular dysfunction, metabolic reprogramming, and cellular injury [6]. Despite the extensive research conducted in this regard, the diagnostic and therapeutic importance of the physiological changes observed in SA-AKI remains poorly understood.

Traditional biomarkers like serum creatinine are commonly employed for diagnosing and predicting AKI in clinical settings; however, their effectiveness is hampered by limited sensitivity and specificity, which can be further complicated by factors such as age, gender, muscle mass, and hydration status [7]. Current clinical treatments for SA-AKI are often reactive and non-specific. Among the newer biomarkers for AKI, neutrophil gelatinase-associated lipocalin (NGAL) has failed to differentiate between patients with or without AKI in the presence of sepsis [8,9], while the kidney injury molecule-1 (KIM-1) has not been thoroughly studied in the context of SA-AKI [10]. Specific biomarkers for diagnosis and effective targets for SA-AKI remain limited [11].

Proteins are essential functional molecules in organisms, and identifying and quantifying large numbers of proteins simultaneously through proteomics is necessary to gain insights into the pathogenesis of diseases. On the other hand, metabolomics is used to quantitatively analyze small molecules in organisms and yields a direct pathophysiological state [12]. With the advancements in mass spectrometry technology, proteomics and metabolomics have emerged as powerful tools for investigating pathogenesis and identifying potential biomarkers [13,14]. It has been reported that acute-phase response proteins are predominantly upregulated in septic mouse kidneys [15]. The plasma proteome of patients with sepsis has shown that endothelial molecules are associated with the development of SA-AKI [16]. The global proteome of mouse kidneys has revealed that kidneys modulate oxidative stress and mitochondrial energetics upon the induction of sepsis [17]. Although there have been single-omic analysis studies of SA-AKI, integrated multi-omics analysis still needs to be improved [18]. Integrating proteomics and metabolomics analyses could allow researchers to draw a multidimensional map of SA-AKI [19].

Herein, we integrated proteomics and metabolomics analysis approaches to provide a relatively comprehensive landscape of SA-AKI with mutual validation. This multi-omics analysis of septic kidneys applied various bioinformatic analyses, including gene set enrichment analysis (GSEA), weighted gene co-expression network analysis (WGCNA), protein and protein interactions (PPI), and MetaboAnalyst analysis. Our study revealed evidence of mitochondrial dysfunction, metabolic disorders, an activated immune response, and the accumulation of catabolic products from polyamines in septic kidneys. Our study offers valuable insights into the pathogenesis of SA-AKI along with potential diagnostic and therapeutic targets.

## 2. Materials and Methods

### 2.1. Animal Model

Male wild-type C57BL6 mice, aged 6–8 weeks and weighing 20–25 g, were obtained from Beijing Vital River Laboratory Animal Technology Company. The mice were housed at the Peking Union Medical College Hospital animal center in a temperature-controlled room (22 °C) with a 12 h light/dark cycle. After acclimatizing for 1 week, the animals were randomized into two groups (*n* = 5) to create the SA-AKI models via the intraperitoneal injection of 9 mg/kg of lipopolysaccharide (LPS, *E. coli* O111:B4, Sigma, St. Louis, MO, USA) and the control mice injected with 0.9% saline. After 24 h, the mice were euthanized to collect blood and kidney samples. All animal experiments were conducted following the National Institutes of Health Guide for the Care and Use of Laboratory Animals and approved by the Ethics Committee of the Peking Union Medical College Hospital (NO. XHDW-2022-017, 16 March 2022).

### 2.2. Analysis of Renal Function

The renal function markers of serum creatinine and blood urea nitrogen (BUN) were assessed using a commercial assay kit produced by Nanjing Jiancheng Bioengineering Institute (Nanjing, China). Briefly, the samples were mixed with reagent A and incubated in a 37 °C water bath for 5 min (or 10 min for BUN), and the absorbance, A1, was recorded at 546 nm (or 640 nm for BUN); then, reagent B was added to the reaction mixture and incubated in a 37 °C water bath for 5 min (or 10 min for BUN) to record the absorbance (A2). The serum creatinine and BUN values were calculated according to the manufacturer’s instructions.

### 2.3. Quantitative Real-Time PCR (qPCR)

Total RNA was extracted from the kidneys using Trizol reagent (Invitrogen, Waltham, MA, USA). Next, total RNA was used to synthesize cDNA with a reverse transcription kit (Vazyme, Nanjing, China). qPCR was carried out on a CFX96 RT-PCR system (Bio-Rad, Hercules, CA, USA) with SYBR Green dye (Vazyme, Nanjing, China). *Actb* was the internal control, and primers were from Primer-Blast [20], as listed in Table S1.

### 2.4. Proteomics Analysis

Proteins were extracted from 10 mg of kidney tissue using an 8 M urea solution. The tissue was then homogenized five times at 4 °C, employing a tissue grinder with a frequency of 60 Hz for each 15 s run, followed by a 10 s pause. Subsequently, centrifugation was performed to collect the supernatants. The protein concentrations of the samples were measured using a BCA protein assay kit. In-solution digestion was carried out by reducing 100 µg of protein using 5 mM of dithiothreitol (DTT) and, subsequently, alkylating the resulting product with 12.5 mM of iodoacetamide (IAM) away from a light source. The proteins were diluted and digested with trypsin (Promega, Madison, WI, USA) for 16 h at 37 °C. Following desalination, tryptic peptides were labeled with tandem mass tags (TMT) 10-plex reagents (Thermo Fisher Scientific, Waltham, MA, USA). TMT-labeled peptides were combined, desalted and then separated using a UPLC system (Thermo Fisher Scientific, Waltham, MA, USA). The fractions were dissolved in formic acid (FA) and analyzed using Liquid Chromatography-Tandem Mass Spectrometry (LC-MS/MS).

### 2.5. LC-MS/MS Analysis

Peptides were separated using a high-performance liquid chromatography (HPLC) system coupled with a Q Exactive HFX mass spectrometer (Thermo Fisher Scientific, Waltham, MA, USA) in the data-dependent acquisition mode. The parameters were as follows: a single full-scan mass spectrum was generated using Orbitrap (300–1800 *m*/*z*; resolution of 60,000), with an automatic gain control (AGC) target of 3 × 10^6^; the MS/MS spectra acquisition settings corresponded to 45,000 for resolution with an AGC target of 1 × 10^5^ and a maximum injection time of 100 ms; the isolation window width was 0.4 Da; and the normalized collision energy for dissociation was 35%.

### 2.6. Peptide and Protein Identification

Relative protein quantification was performed using the Proteome Discoverer (PD) 2.1 software (Thermo Fisher Scientific, Waltham, MA, USA), which accessed the UniProt mouse database. The searching process used the following criteria: full tryptic specificity was required, a tolerance of two missed cleavages was set, carbamidomethylation and TMT 10-plex were fixed modifications, and variable modification corresponded to oxidation. The searched data were further processed using the percolator function in Proteome Discoverer to allow for filtering with a 1% peptide false discovery rate (FDR). Relative protein quantification was carried out using PD 2.1 according to the intensities of reporter ions per peptide. The MS proteomics data are available in the ProteomeXchange [21] library via the PRIDE repository [22] under the identifier PXD044371. All proteins identified and the relative abundance values are listed in Table S2.

### 2.7. Metabolomics Analysis

A total of 20 mg of renal tissue was homogenized in pre-chilled 80% methanol five times using a tissue grinder at 4 °C, with each run lasting 15 s at a frequency of 60 Hz, followed by a 10 s pause. Subsequently, the homogenates were centrifuged to collect the supernatants. Firstly, the metabolites were wholly dried with a lyophilizer. Then, dissolved metabolites were analyzed using LC-MS/MS. The profiling of targeted and untargeted metabolites was conducted using a TSQ Quantiva™ Triple Quadrupole Mass Spectrometer and a Q-Exactive Mass Spectrometer (Thermo Fisher Scientific, Waltham, MA, USA), respectively. Metabolites were identified based on retention time and quantitated using Trace-Finder 3.2 (Thermo Fisher Scientific, Waltham, MA, USA). For untargeted profiling, metabolites were identified based on MS/MS matching with the standard library. Two levels of identification were achieved in the analysis, one of which was through MS/MS confirmation and the other via potential assignment according to precursor ion masses. Missing values were imputed using the mean imputation method. The MS metabolomics data are available at www.ebi.ac.uk/metabolights/MTBLS8350 [23]. All metabolites identified and the relative abundance values are listed in Table S3.

### 2.8. Statistical Methods and Bioinformatics Analysis

Statistical analysis was conducted using GraphPad Prism 9.1.2 software (GraphPad, La Jolla, CA, USA). Approximately normal distribution of relative abundance was confirmed, and differences between two groups were analyzed using a two-tailed Student’s *t*-test. Multiple testing was performed using the Benjamini–Hochberg procedure. The threshold for statistical significance was a *p*-value of less than 0.05. The STRING database was utilized, for which a high confidence score of 0.9 was set as the cut-off value to generate protein–protein interaction (PPI) networks [24]. The constructed networks were visualized using Cytoscape 3.9.1 [25,26]. GSEA was performed based on the Gene Ontology (GO) and Kyoto Encyclopedia of Genes and Genomes (KEGG) databases using the “clusterProfiler v4.6.2” R package [27]. GOChord, GOHeat, and GOCluster plots were visualized using the “GOplot” R package [28]. Weighted gene co-expression network analysis (WGCNA) was performed and visualized by the “WGCNA” R package [29]. Metabolite set enrichment analysis and multivariate exploratory ROC analysis were carried out using MetaboAnalyst 5.0 [30,31].

## 3. Results

### 3.1. The Global Proteomic View of the Septic Kidneys

**Figure 1:**
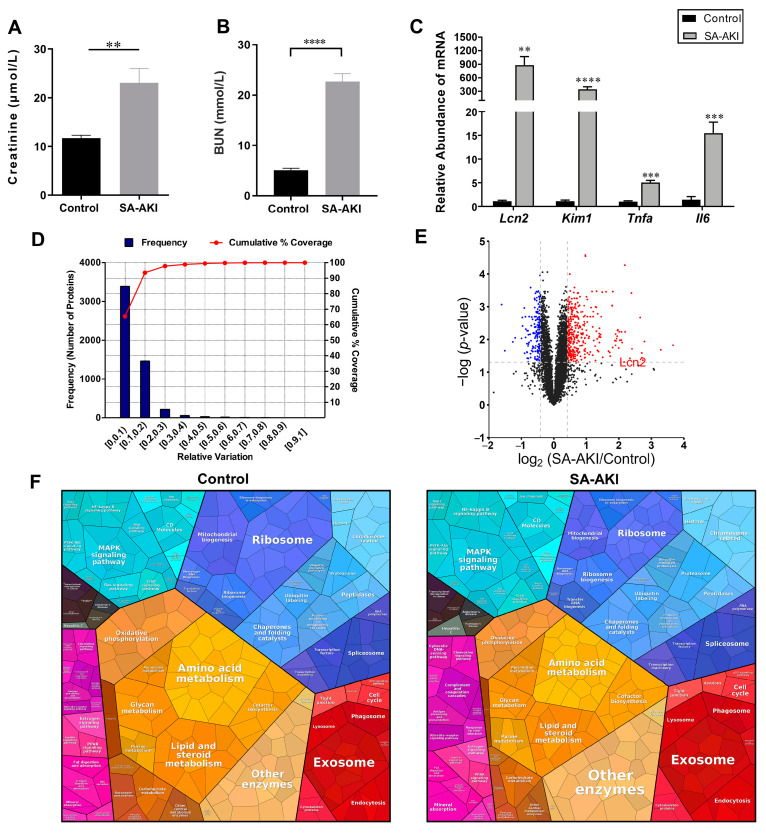
A global proteomic view of the septic kidneys. (**A**) Serum creatinine and (**B**) blood urea nitrogen (BUN) levels in the sepsis-associated acute kidney injury (SA-AKI) mice compared with the control mice (*n* = 5; mean ± SEM). (**C**) mRNA expression of *Lcn2*, *Kim1*, *Tnfa*, and *Il6* in SA-AKI kidney tissue compared to that of the control kidneys (*n* = 5; mean ± SEM). (**D**) Experimental variations of proteomics analysis of kidney tissue in mice with SA-AKI and control mice. (**E**) A volcano plot was created to visualize the differences in the protein expression levels between the kidney tissues of the SA-AKI and control mice, with blue and red dots representing downregulated and upregulated proteins, respectively. The proteins exhibiting significant differences (*p*-value < 0.05; ratios of ≤0.75 or ≥1.33) are highlighted. (**F**) Proteomaps of SA-AKI kidney tissue compared with the control group. The polygons in proteomaps consist of proteins, with the size representing protein abundance. Proteins belonging to the same category are placed in adjacent locations [33]. **** *p* < 0.0001, *** *p* < 0.001, and ** *p* < 0.01.

We created an SA-AKI mouse model with LPS stimulation, which significantly increased serum creatinine and BUN levels after 24 h of treatment (Figure 1A,B). We investigated the mRNA expression levels of several kidney injury marker genes and found that neutrophil gelatinase-associated lipocalin protein (*Lcn2*) and kidney injury molecule-1 (*Kim-1*) levels were significantly higher in the SA-AKI kidneys; we also found elevated expression of tumor necrosis factor (*TNFa*) and interleukin-6 (*Il6*) (Figure 1C). To further elucidate the molecular mechanisms of SA-AKI, we carried out a quantitative proteomics analysis to characterize the proteome changes of renal tissue after SA-AKI. A total of 5185 proteins were identified, for which there was a less than 1% false discovery rate (FDR) (Table S2). A threshold cut-off was established using percentage variations corresponding to 88% coverage [32]. Proteins exhibiting ratios of ≥1.33 or ≤0.75 and *p*-values < 0.05 compared to the controls were considered upregulated or downregulated, respectively. There were 353 upregulated proteins and 166 downregulated proteins in the SA-AKI tissue compared with the control tissue (Figure 1D,E). We then constructed proteomaps to visually cluster the differentially expressed proteins according to their KEGG pathway annotations to highlight proteome composition conservation after SA-AKI [33]. The proportions of organismal system categories (such as complement and coagulation cascades, antigen presentation, and the cytosolic DNA-sensing pathway) and environmental-information-processing categories (such as the nuclear factor-kB pathway and the cluster of differentiation molecules) were higher in the SA-AKI group. In contrast, the changes in metabolic categories were prominent. The SA-AKI group had lower proportions of proteins related to oxidative phosphorylation, lipid metabolism, amino acid metabolism, and glycan metabolism. Along with the metabolic changes, we also observed lower levels of mitochondrial biogenesis and ribosome proteins in the SA-AKI group (Figure 1F). The results regarding proteome composition conservation indicated that there were dynamic changes after septic injury.

### 3.2. Septic Injury Impaired Mitochondrial Function in Kidneys

**Figure 2:**
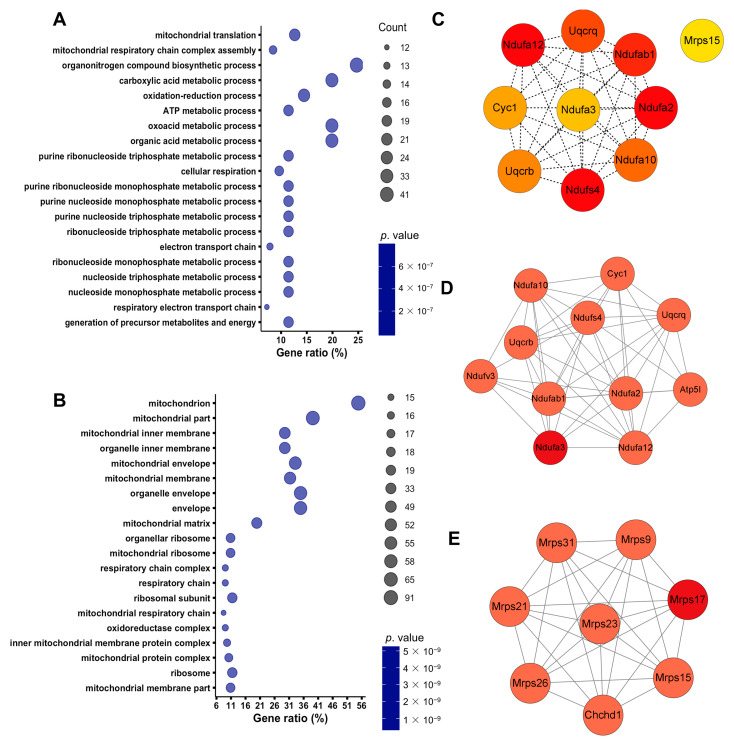
Mitochondrial homeostasis disordered in SA-AKI kidneys. (**A**) The biological process and (**B**) cellular component enrichment of downregulated proteins in the SA-AKI kidneys analyzed by Gene Ontology (GO) with DAVID 2021. (**C**) A total of 10 hub proteins were identified by Cytohubba via the maximal clique centrality method (Red represents a higher score, while yellow represents a lower score). (**D**,**E**) Top two PPI (protein and protein interaction) networks analyzed by MCODE. The first cluster had 11 nodes and 47 edges (score = 9.4). The second cluster had 8 nodes and 47 edges (score = 8).

To further understand the proteomic changes in the SA-AKI kidneys, we performed a GO analysis of the differentially expressed proteins using DAVID 2021 [34]. The biological process enrichment result showed that downregulated proteins in the SA-AKI kidneys were primarily involved in mitochondrial functions such as mitochondrial translation; mitochondrial respiratory chain complex assembly; energy metabolism processes including ATP metabolic process, oxidation–reduction process, cellular respiration, and purine and nucleoside metabolism (Figure 2A). Most remarkably, cellular component ontology analysis showed that downregulated proteins were mainly enriched in mitochondrion proteins, respiratory chain complex, and oxidoreductase complex (Figure 2B), with nearly 67% of the downregulated organelle proteins being enriched in mitochondria (Figure S1). To further investigate the interactions between the differentially expressed proteins, we constructed PPI networks using STRING [24]. We then utilized the Cytohubba and Molecular Complex Detection (MCODE) plug-in of Cytoscape 3.9.1 to find hub modules in the PPI networks [25,26]. The top 10 hub proteins identified using Cytohubba were mainly involved in the mitochondrial electron transport chain (Figure 2C). MCODE analysis revealed that the top two PPI networks were mainly composed of mitochondrial-respiratory-chain-related proteins and mitochondrial ribosomal proteins (Figure 2D,E). Our findings suggested that septic infection disrupted mitochondrial homeostasis in the kidneys, which is consistent with previous research findings [17].

**Figure 3:**
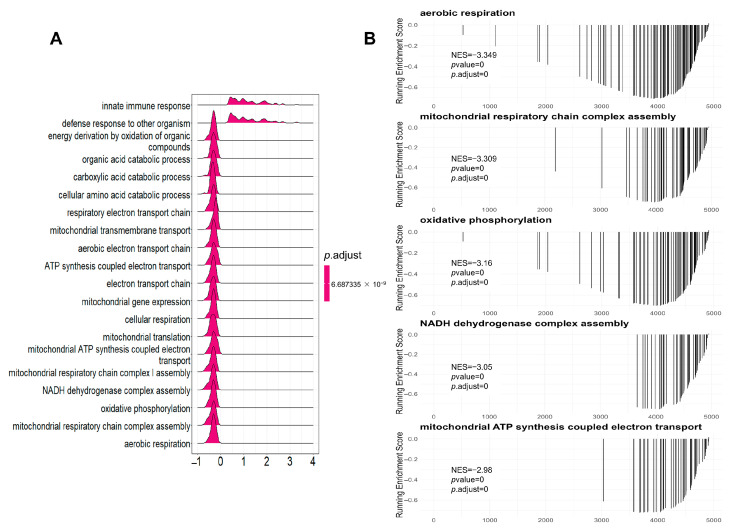
GSEA (gene set enrichment analysis) analysis of all identified proteins in the proteomics analysis of the SA-AKI kidneys compared with the control group based on the GO database. (**A**)The top 20 pathways with significant differences in the GO database. (**B**) The top five pathways according to the GSEA normalized enrichment score in the GO database.

Gene set enrichment analysis (GSEA) is a powerful method that allows for the identification of coordinated changes in groups of genes, which can identify subtle but coordinated changes in gene expression that may be missed by other methods [35,36]. We conducted a GSEA analysis of all of the identified proteins by clusterProfiler v4.6.2 [27]. Our analysis showed that the top 20 pathways with significant differences in the GO database mainly concerned mitochondrial respiratory chain complex assembly, energy derivation by the oxidation of organic compounds, the innate immune response, and defense responses against other organisms (Figure 3A). Specifically, ATP synthesis-coupled electron transport, cellular respiration, mitochondrial respiratory chain complex assembly, NADH dehydrogenase complex assembly, and oxidative phosphorylation pathways were significantly downregulated. The top five pathways, according to the GSEA normalized enrichment score (NES), were aerobic respiration, mitochondrial respiratory chain complex assembly, oxidative phosphorylation, NADH dehydrogenase complex assembly, and mitochondrial ATP synthesis coupled electron transport (Figure 3B). All five pathways were significantly downregulated, and the cneplot revealed shared genes between the top five enrichment pathways (Figure S2). Consistent with the GO analysis, the GSEA analysis using the KEGG database also showed significant downregulation of the oxidative phosphorylation, citrate cycle, and fatty acid metabolism pathways (Figure S3A–C). These results provided strong evidence of mitochondrial dysfunction in sepsis renal injury. In addition, we found that the peroxisome pathway, which is essential for the turnover of complex lipids and reactive species, was downregulated (Figure S3D). Peroxisomes have been identified as regulators of oxidative stress during infection [37]. Therefore, downregulated peroxisome metabolism may contribute to oxidative stress and thus aggravate renal injury.

### 3.3. Immune-Related Pathways Were Significantly Activated in SA-AKI Kidneys

**Figure 4:**
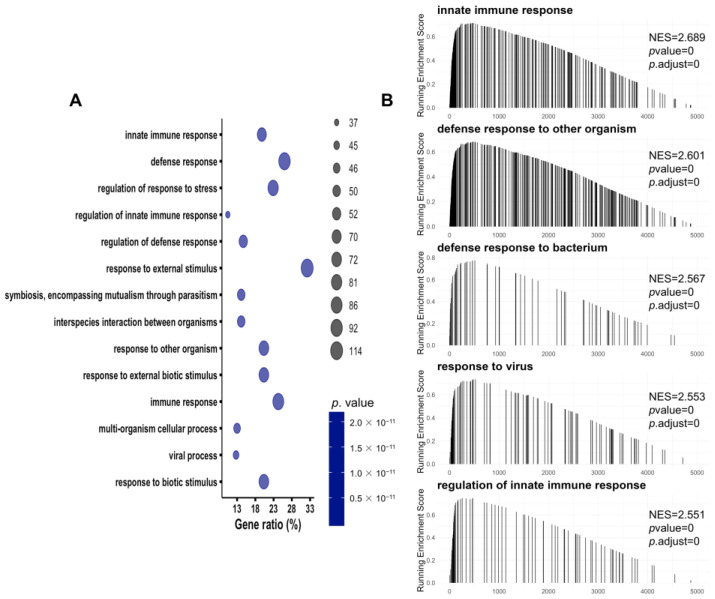
Function enrichment analysis of upregulated proteins in the SA-AKI kidneys. (**A**) The biological process enrichment of upregulated proteins in the SA-AKI kidneys analyzed by GO with DAVID 2021. (**B**) The top five upregulated pathways according to the GSEA normalized enrichment score in the GO database.

To investigate the upregulated proteins in the SA-AKI kidneys, we performed biological process enrichment via GO analysis. The results showed that immune-related pathways were significantly enriched in the SA-AKI kidneys (Figure 4A), with the top 12 terms determined according to the GO z-score and visualized using the GOChord, GOHeat, and GOCluster plots (Figure S4) [28]. Consistent with the GO analysis, the GSEA analysis revealed that the top five upregulated pathways in the GO database were the innate immune response and defense responses to other organisms, such as bacteria and viruses (Figure 4B). The top five upregulated pathways in the KEGG database included complement and coagulation cascades, herpes simplex virus-1 infection, Epstein–Barr virus infection, antigen processing and presentation, and phagosomes (Figure S5). Immune pathways were activated to adapt to the infection conditions upon septic injury in the kidneys.

### 3.4. Construction of SA-AKI Protein Co-Expression Networks

**Figure 5:**
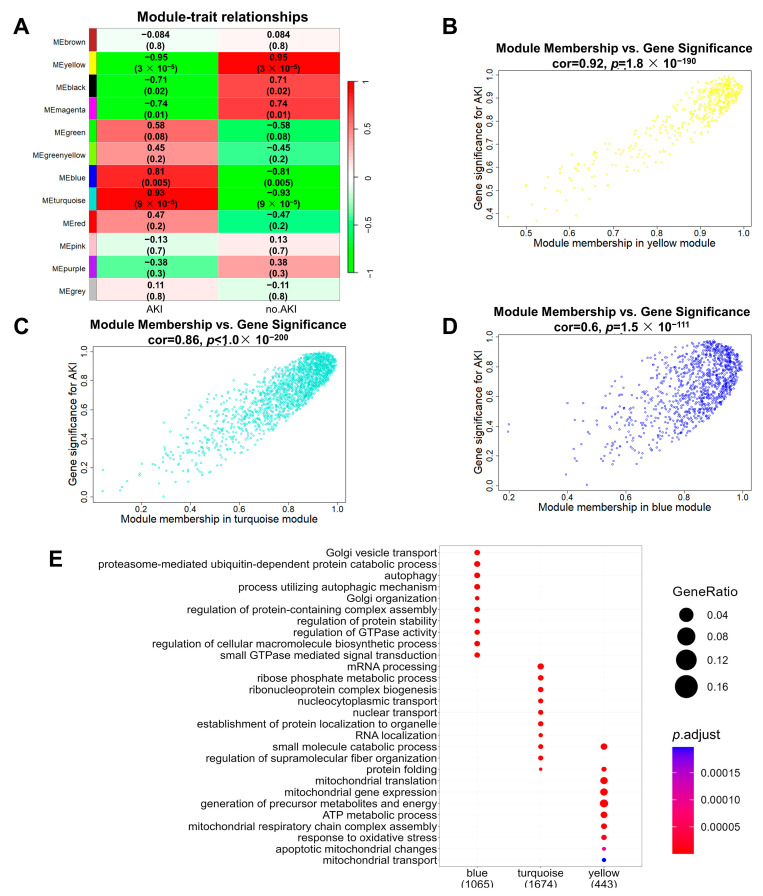
The co-expression network of proteins in SA-AKI kidneys constructed by weighted gene co-expression network analysis. (**A**) Heatmap illustrates the relationships between modules and the SA-AKI trait. Each row corresponds to a module, and each cell contains the correlation as well as the corresponding *p*-value in the bracket. (**B**–**D**) A scatterplot of module membership vs. gene significance with respect to SA-AKI in the yellow, turquoise, and blue modules. Gene significance (the absolute value) represents associations of individual genes with the SA-AKI trait. Module membership represents the correlation between each module and the gene expression profile. (**E**) GO analyses of the proteins in yellow, turquoise, and blue modules.

Using the weighted gene co-expression network analysis (WGCNA) one-step network construction method, we obtained 12 co-expression modules of all of the identified proteins in the proteomics data of the SA-AKI kidneys compared to the control group (Figure 5A and Figure S6). These co-expression modules are clusters of genes with robust absolute correlations, identified through unsupervised clustering methods within the WGCNA framework. Co-expression modules play a pivotal role in identifying crucial genes or pathways associated with specific biological processes or diseases [38]. Module–trait relationship analysis showed that the yellow module had the most negative correlation with SA-AKI (Figure 5A), while the turquoise and blue modules had substantial correlations with SA-AKI. Further analysis quantified the correlations between module membership (MM) and gene significance (GS) in the three modules. Specifically, the correlations between module membership and gene significance in the yellow, turquoise, and blue modules were 0.92, 0.86, and 0.6, respectively (Figure 5B–D). The high correlation between GS and MM in a given module illustrates that proteins highly associated with SA-AKI are also pivotal elements of that module. This indicated that the proteins in these three modules were important elements associated with SA-AKI. Consequently, we performed GO analyses of the proteins found in the yellow, turquoise, and blue modules (Figure 5E). The proteins in the yellow module, i.e., the module that was negatively correlated with SA-AKI, were mainly enriched in mitochondria-related processes, indicating that mitochondrial dysfunction was an incredibly crucial facilitator for the development of SA-AKI. The correlation between module membership and gene significance in the yellow module was the highest, further supporting the importance of mitochondrial dysfunction in relation to SA-AKI. The proteins in the turquoise module were mainly involved in mRNA processing, ribose phosphate metabolism, ribonucleoprotein complex biogenesis, nucleocytoplasmic transport, nuclear transport, etc. The proteins in the blue module were mainly related to protein stability, such as Golgi vesicle transport, proteasome-mediated ubiquitin-dependent protein catabolic processes, autophagy, and the regulation of protein-containing complex assembly.

### 3.5. SA-AKI Induced Metabolic Disorders

**Figure 6:**
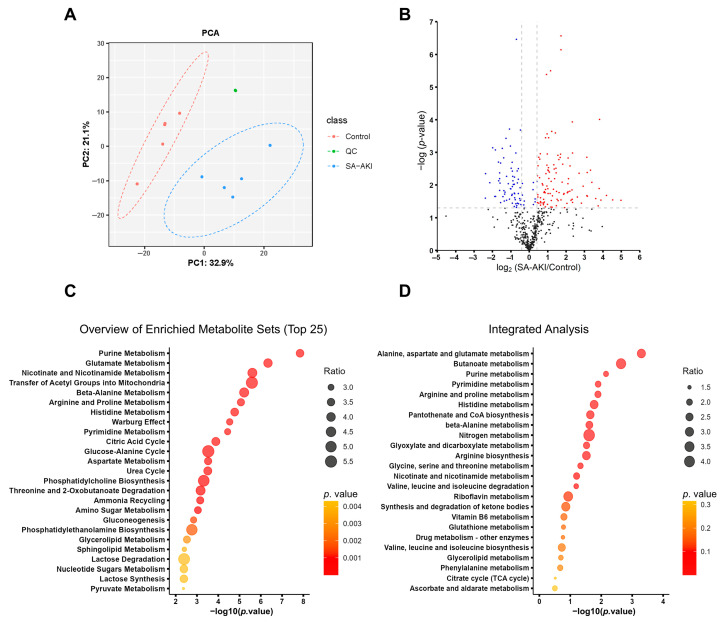
Metabolomic profiling of the septic kidneys. (**A**) Principal component analysis (PCA) of metabolites showed that metabolites in SA-AKI and control kidneys were different. QC, quality control. (**B**) A volcano plot was generated to visualize the differences in metabolite levels between SA-AKI kidney tissue and control kidney tissue, with blue and red dots representing downregulated and upregulated metabolites, respectively. The metabolites exhibiting significant differences (*p*-value < 0.05; ratios of ≤0.75 or ≥1.33) were highlighted. (**C**) The top 25 enriched metabolite sets were analyzed by MetaboAnalyst 5.0. (**D**) The integrated analysis of proteomics and metabolomics data was performed by MetaboAnalyst 5.0.

We conducted metabolomics analysis on five biological replicates to comprehensively profile SA-AKI and identify key metabolites associated with the condition. Principal component analysis (PCA) identified the differences between the metabolites of the SA-AKI and control kidneys (Figure 6A). Metabolites exhibiting ratios of ≥1.33 or ≤0.75 and *p*-values < 0.05 compared to the controls were considered upregulated or downregulated, respectively. The SA-AKI group displayed a rise in 111 metabolite levels and a reduction in 66 metabolites (Figure 6B). To elucidate the underlying metabolic disruptions of SA-AKI, we conducted an enrichment analysis using MetaboAnalyst 5.0. The results pointed to significant disturbances in central carbon metabolism, including processes such as the transfer of acetyl groups into mitochondria, the Warburg effect, and the citric acid cycle. Moreover, amino acid metabolism, nucleotide metabolism, as well as nicotinate and nicotinamide metabolism were also notably impacted, as demonstrated in Figure 6C. In an effort to elucidate the intricate connections between the proteins and metabolites associated with SA-AKI, we performed an integrated analysis of proteomics and metabolomics data using MetaboAnalyst. This analysis uncovered enriched metabolic pathways primarily centered around amino acid metabolism, including pathways such as alanine, aspartate, and glutamate metabolism, as well as arginine and proline metabolism, and histidine metabolism. Additionally, there were significant enrichments observed in nucleotide metabolism, butanoate metabolism, pantothenate and CoA biosynthesis, and glyoxylate and dicarboxylate metabolism, as illustrated in Figure 6D.

**Figure 7:**
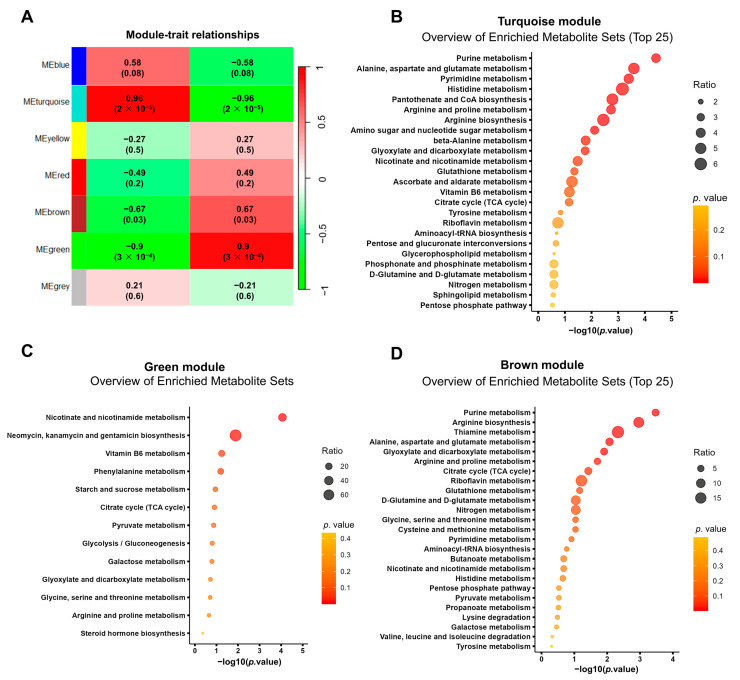
The co-expression network of metabolites in SA-AKI kidneys was constructed by weighted gene co-expression network analysis. (**A**) Heatmap shows module–SA-AKI trait associations. Each row corresponds to a module, and each cell contains the correlation as well as the corresponding *p*-value in the bracket. (**B**–**D**) Enrichment analysis with MetaboAnalyst 5.0 of the metabolites in turquoise, green, and brown modules.

We constructed a metabolite co-expression network using weighted gene co-expression network analysis (WGCNA) and identified seven co-expression modules, among which the turquoise, green, and brown modules were further analyzed (Figure 7A). Enrichment analysis via MetaboAnalyst revealed that the metabolites in the turquoise module were most enriched in nucleotide metabolism, amino acid metabolism, glyoxylate and dicarboxylate metabolism, nicotinate and nicotinamide metabolism, and glutathione metabolism (Figure 7B). Notably, nicotinate and nicotinamide metabolism were significantly enriched in the green module (Figure 7C). Consistent with the turquoise module, purine and amino acid metabolism were enriched in the brown module. In addition, thiamine metabolism and citrate cycle were also enriched in the brown module (Figure 7D). These metabolic disorders were closely related to SA-AKI and could serve as potential biomarkers.

### 3.6. Catabolic Products of Polyamines Accumulated in SA-AKI Kidneys

**Figure 8:**
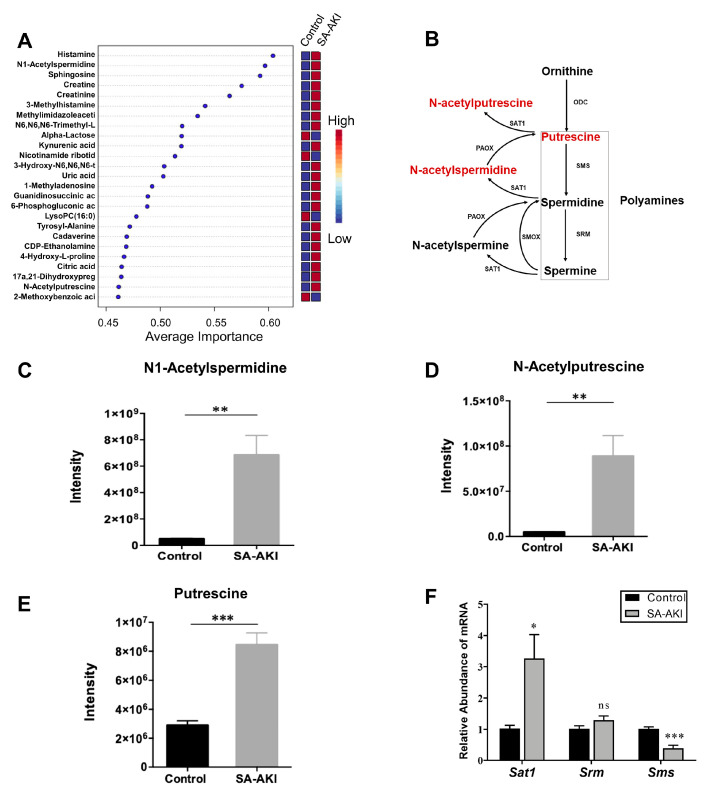
Catabolic products of polyamines increased in septic kidneys. (**A**) Multivariate exploratory receiver operating characteristic (ROC) analysis was performed with MetaboAnalyst 5.0. Candidate biomarker metabolites were ranked according to their average importance scores. (**B**) Schematic graph of polyamine catabolism. ODC, ornithine decarboxylase; SMS, spermine synthase; SRM, spermidine synthase; SAT1, diamine acetyltransferase 1; SMOX, spermine oxidase; PAOX, peroxisomal N (1)-acetyl-spermine/spermidine oxidase. Metabolites in red fonts represent those upregulated in the SA-AKI kidneys. (**C**–**E**) Metabolite intensity of N1-acetylspermidine, N-acetylputrescine, and putrescine in the SA-AKI and control kidneys. (**F**) mRNA expression of *Sat1*, *Srm*, and *Sms* in SA-AKI kidney tissue compared with the control group. *n* = 5, mean ± SEM; *** *p* < 0.001, ** *p* < 0.01, and * *p* < 0.05; ns: no significance.

Metabolomics and multivariate exploratory receiver operating characteristic (ROC) analysis showed that N-acetylspermidine and N-acetylputrescine had high average importance scores as vital features of SA-AKI (Figure 8A). Via polyamine catabolism, N-acetylspermidine can be converted back to putrescine and then further catalyzed by SAT1 to generate N-acetylputrescine (Figure 8B). Both the N-acetylspermidine and N-acetylputrescine levels increased more than 10 times with upregulated putrescine (Figure 8C–E). Moreover, the mRNA expression of *Sat1*, the rate-limiting enzyme of polyamine catabolism, increased in the SA-AKI kidneys (Figure 8F). These results showed that the catabolic products of polyamines accumulated in the SA-AKI kidneys.

## 4. Discussion

SA-AKI is a common and severe complication of sepsis characterized by acute kidney insufficiency that accounts for 41% of mortality in ICUs; this figure was especially high during the coronavirus disease 2019 (COVID-19) pandemic [11,39]. Besides serum creatinine and urine output, no well-accepted diagnostic marker of SA-AKI has been determined despite decades of research, thus limiting early diagnosis and treatment. However, proteomics and metabolomics have provided a possibility of identifying novel biomarkers and potential targeted therapies with which to improve the outcomes of patients afflicted by this dangerous condition.

In this study, we carried out proteomics and metabolomics analyses to gain a broader landscape of SA-AKI. Our results demonstrated that septic kidneys exhibited a decrease in the proportions of proteins related to oxidative phosphorylation, lipid metabolism, amino acid metabolism, and glycan metabolism. Furthermore, our GO enrichment and GSEA analyses revealed disrupted mitochondrial homeostasis in septic kidneys. Additionally, we identified 10 hub proteins within the PPI networks of differentially expressed proteins primarily involved in the mitochondrial respiratory chain and mitochondrial ribosomal proteins. Both proteomics and metabolomics weighted gene co-expression network analyses (WGCNAs) revealed a significant enrichment of mitochondrial proteins and related metabolites. Our multi-omics approach provided an integrated and simultaneously derived profile ranging from proteins to metabolites in septic kidneys, highlighting mitochondrial dysfunction as a crucial pathophysiological hallmark of SA-AKI. The kidneys have high densities of mitochondria, facilitating the dynamic process of actively reabsorbing nutrients and electrolytes [40]. The priority shift in energy utilization during sepsis is considered an evolutionarily conservative defense response that limits non-essential functions to avoid excessive energy consumption [41]. However, persistent decreased oxidative phosphorylation also leads to the cell cycle arrest of tubular epithelial cells (TECs), progressing to a risk factor for organ damage after acute reactions [42]. Our previous study additionally demonstrated that renal injury can be exacerbated by impaired mitochondrial function in tubular epithelial cells [43]. Thus, the identified 10 hub proteins related to mitochondria in the SA-AKI kidneys might provide clues with regard to finding potential therapeutic targets.

The accumulation of the catabolic products of polyamines (N-acetylspermidine, N-acetylputrescine, and putrescine) with an increased mRNA level of *Sat1* indicated that there was enhanced polyamine catabolism in the septic kidneys. Unraveling the implications of catabolic products derived from polyamines in SA-AKI remains a relatively uncharted area. The role of polyamine catabolism is involved in diverse cellular processes, including gene expression, protein synthesis, and oxidative stress regulation [44], as well as the genesis of disorders like cerebral ischemia and acute liver injury [45,46]. Recent studies have even highlighted increased levels of acetylpolyamine in the context of COVID-19 and trauma-related infections [47,48], further supporting the potential utility of polyamine catabolic products as non-invasive diagnostic markers. Moreover, the polyamine spermidine has been linked to enhanced mitochondrial respiratory function via eIF5A hypusination [49,50,51]. We speculate that the enhanced polyamine catabolism and subsequent reduction in spermidine might impair mitochondrial function in SA-AKI. The accumulation of the catabolic products of polyamines has been less well studied in SA-AKI, and our study provides new insights into this area. The intricacies of polyamine catabolism in SA-AKI, however, remain elusive and warrant in-depth exploration.

The MetaboAnalyst enrichment analysis also revealed significant enrichment in nucleotide metabolism, amino acid metabolism, glyoxylate metabolism, dicarboxylate metabolism, and glutathione metabolism, with a remarkable enrichment in nicotinate and nicotinamide metabolism in SA-AKI. Since nicotinamide (NAM) and nicotinamide adenine dinucleotide (NAD) act as core effectors in protecting against oxidative stress and preventing kidney ischemia/reperfusion (I/R) injury [52], they could also serve as a potential therapeutic target for SA-AKI; in this regard, the renoprotective effect of nicotinamide (NAM) supplementation against acidosis-induced acute kidney injury has been reported [53]. Together, these findings have important therapeutic implications for SA-AKI.

The weighted gene co-expression network analysis of proteomics revealed that the turquoise module was related to mRNA processing and stability. Existing research indicates that some bacteria can manipulate host gene expression, mRNA procession, and mRNA stability to modulate innate immune response [54]. Similarly, the blue module’s association with protein stability aligns with the findings that viral infections like severe acute respiratory syndrome coronavirus 2 (SARS-CoV-2) can influence host protein stability [55,56]. Our results draw attention to the roles of RNA and protein stability in the context of SA-AKI. Nonetheless, an in-depth exploration is indispensable to explore the precise regulatory mechanisms underlying this phenomenon, potentially offering novel avenues for intervention. An integrated analysis of proteins and metabolites suggested that amino acid metabolism was mainly enriched, which is consistent with characteristics of septic patients’ serum samples [19]. Amino acid metabolism has been found to be a pivotal regulator of innate and adaptive immunity [57,58]. These data suggested that characteristic proteins and metabolites related to the immune response could serve as potential therapeutic targets for SA-AKI.

## 5. Study Limitations

Our study offers a multidimensional overview of SA-AKI derived directly from septic kidney tissue. Our data broadly agree with previous discoveries in the context of mitochondrial dysfunction [17]. Moreover, we observed an increase in the levels of the catabolic products of polyamines, an area less explored in S-AKI research. However, the current study could be more flawless. One of its limitations is the small specimen encompassing only five mice from a single animal model. A larger specimen and finer grouping can be used to investigate changes in the early and late phases of SA-AKI in future work. Additionally, more mouse models of SA-AKI (such as cecal ligation puncture) and human samples are needed to increase generalizability. Obtaining human kidney samples for AKI proves to be challenging, with fluid samples predominantly used due to their accessibility. This underlines the indispensable role of murine models in advancing AKI research. Nevertheless, translating insights from murine models to human samples remains a persistent challenge. This emphasizes the necessity for subsequent validation of findings observed in mouse models through human studies. Although the current study identified potential proteins, metabolites, and pathways involved in SA-AKI, experiments conducted in vivo and in vitro are required to investigate the precise molecular mechanisms and validate the individual biomarkers of SA-AKI in further studies. Furthermore, omics techniques employ high-throughput methodologies to provide a relatively comprehensive understanding of disease pathogenesis. However, due to sample complexity, low protein concentrations, and various technical limitations, certain proteins or metabolites might prove challenging to detect or identify. Hence, it remains crucial to complement these outcomes with additional methods for biological validation.

## 6. Conclusions

In summary, multi-omics analysis provides new insights and a multi-faceted understanding of the pathophysiology of SA-AKI with mitochondrial dysfunction, metabolic disorders, the activation of immune-related pathways, and the catabolic products of polyamine accumulation, which might contribute to the development of new diagnostic biomarkers and therapeutic targets of SA-AKI.

## Data Availability

All relevant data of this study are presented. Additional data will be provided upon request.

## Supplementary Materials

The following supporting information can be downloaded at: https://www.mdpi.com/article/10.3390/biom13091329/s1, Table S1. The primers used in this study. Table S2. The list of all proteins identified in the SA-AKI proteome. Table S3. The list of all metabolites identified in the SA-AKI metabolome. Figure S1. Cellular component enrichment analysis of downregulated proteins in SA-AKI kidneys [59]. Figure S2. Cneplot representing shared genes between the top five enrichment pathways in GSEA analysis of all identified proteins based on the GO database. Figure S3. GSEA analysis of all identified proteins in proteomics analysis of SA-AKI kidneys compared with the control group based on the KEGG database. Figure S4. GO plots of upregulated proteins in SA-AKI kidneys [28]. Figure S5. The top five upregulated pathways according to the GSEA normalized enrichment score in the KEGG database. Figure S6. The co-expression network of proteins in SA-AKI kidneys constructed by weighted gene co-expression network analysis (WGCNA).
