# Supplementary material for: Multidimensional Landscape of SA-AKI Revealed by Integrated Proteomics and Metabolomics Analysis
Source: Biomolecules. 2023 Aug 30;13(9):1329. doi: 10.3390/biom13091329 (PMC10526551; doi:10.3390/biom13091329)
Supplement: Supplementary file 1 [file biomolecules-13-01329-s001.zip › Supplementary Figure.pdf]

Multidimensional Landscape of SA-AKI Revealed by Integrated Proteomics and Metabolomics Analysis

Jiatong Xu [1,2], Jiaying Li [3], Yan Li [1], Xiaoxiao Shi [3], Huadong Zhu [1,*] and Limeng Chen [3,*]

[1] Emergency Department, State Key Laboratory of Complex Severe and Rare Diseases, Peking Union Medical College Hospital, Chinese Academy of Medical Science and Peking Union Medical College, Beijing 100730, China
[2] Medical Research Center, Peking Union Medical College Hospital, Chinese Academy of Medical Science and Peking Union Medical College, Beijing 100730, China.
[3] Department of Nephrology, State Key Laboratory of Complex Severe and Rare Diseases, Peking Union Medical College Hospital, Chinese Academy of Medical Science and Peking Union Medical College, Beijing 100730, China
* Correspondence: zhuhd@pumch.ac.cn (H.Z.); chenlimeng@pumch.ac.cn (L.C.)

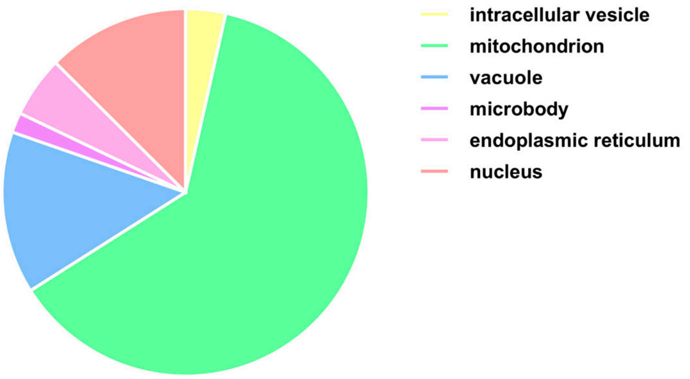

Figure S1. Cellular component enrichment analysis of downregulated proteins in SA-AKI kidneys analyzed by PANTHER 17.0 [59].

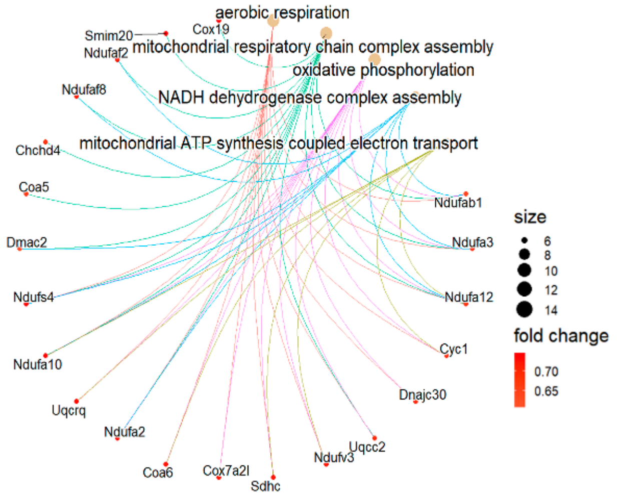

Figure S2. Cneplot representing shared genes between the top five enrichment pathways in GSEA analysis of all identified proteins based on the GO database.

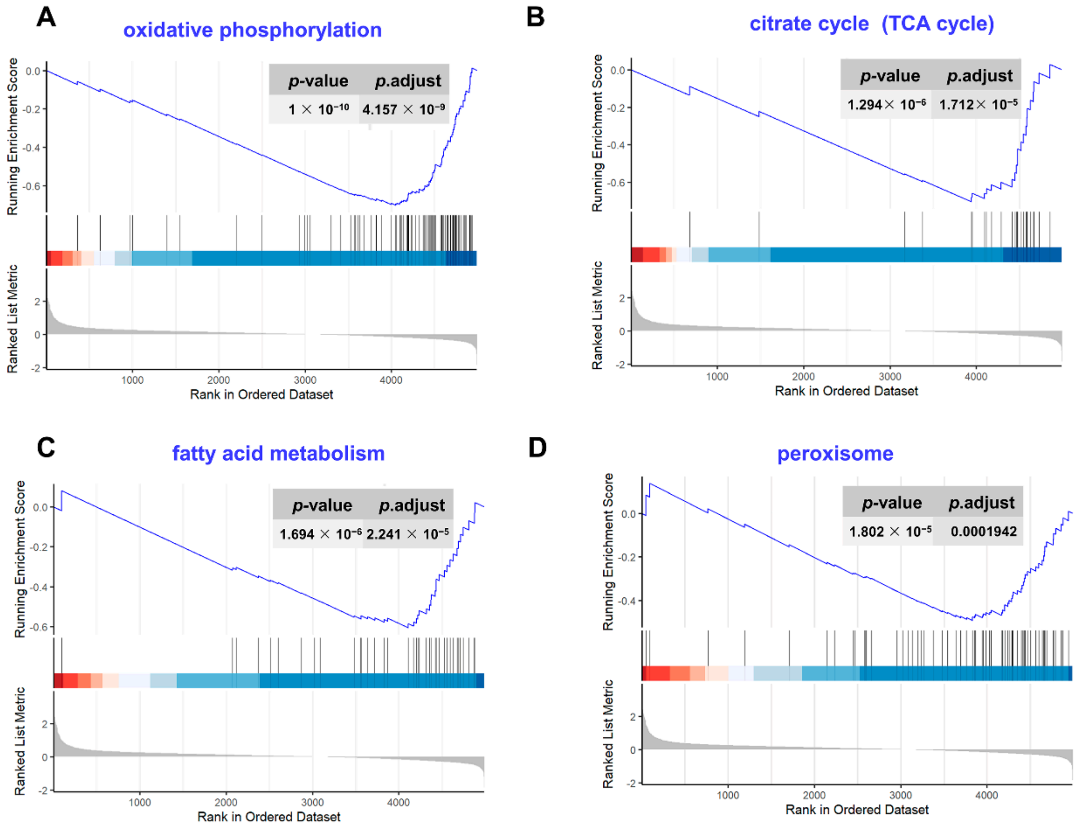

Figure S3. GSEA analysis of all identified proteins in proteomics analysis of SA-AKI kidneys compared with the control group based on the Kyoto Encyclopedia of Genes and Genomes (KEGG) database. (A-D) GSEA plots showed oxidative phosphorylation, citrate cycle, fatty acid metabolism, and peroxisome metabolism were significantly downregulated in the KEGG database.

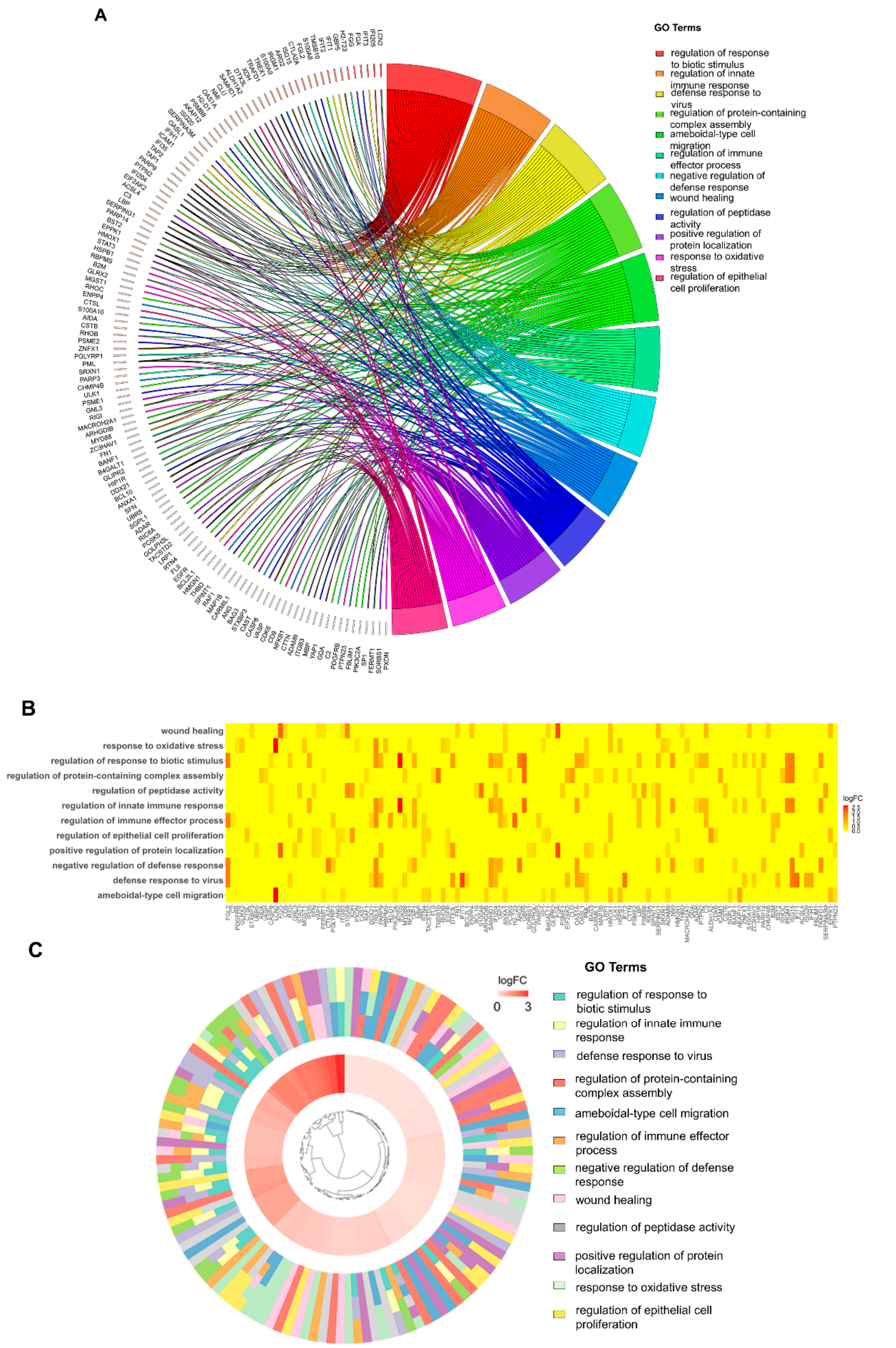

Figure S4. GO plots of upregulated proteins in SA-AKI kidneys. (A) GOChord plot visualized the interaction between the top 12 terms and proteins. GOChord plot was visualized with the R package GOplot [28]. (B) GOHeat plot visualized the interaction between the top 12 terms and proteins. (C) The cluster results of the top 12 terms were visualized by GOCluster plot.

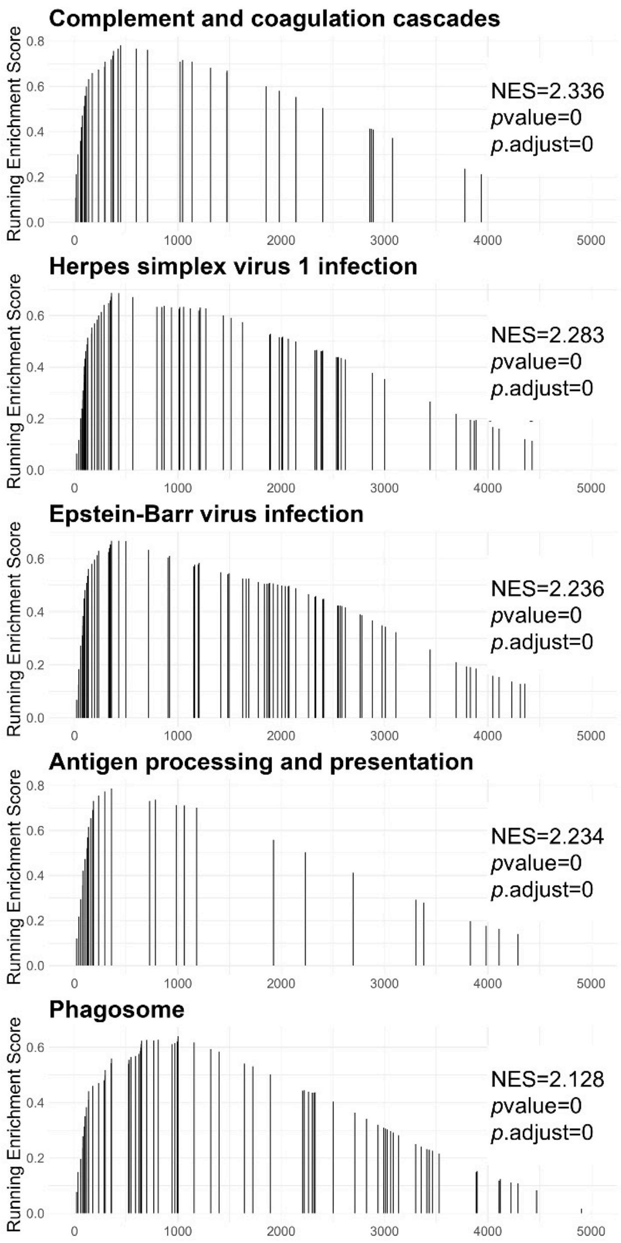

Figure S5. The top five upregulated pathways according to the GSEA normalized enrichment score in the KEGG database.

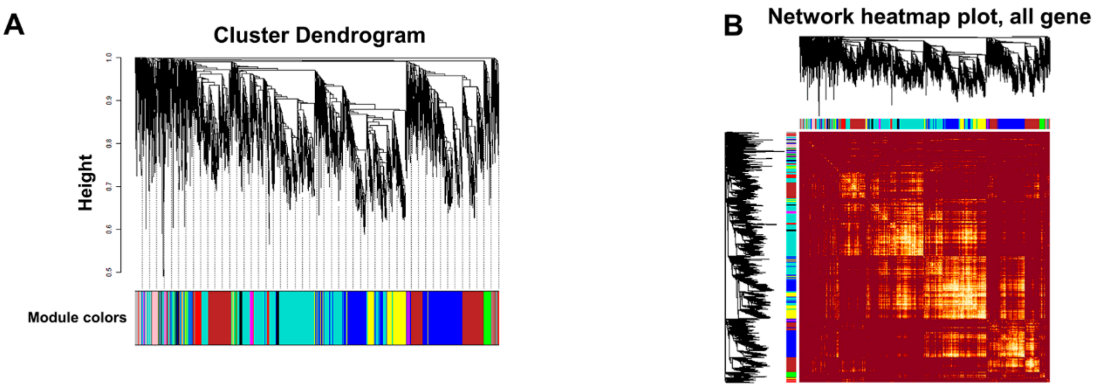

Figure S6. The co-expression network of proteins in SA-AKI kidneys constructed by weighted gene co-expression network analysis (WGCNA). (A) The clustering dendrogram showed 12 protein co-expression modules with dissimilarity based on the topological overlap. (B) The network heatmap plot visualized the protein network and depicted the Topological Overlap Matrix among all proteins.
